# Shotgun metagenomics to investigate unknown viral etiologies of pediatric meningoencephalitis

**DOI:** 10.1371/journal.pone.0296036

**Published:** 2023-12-21

**Authors:** Andrea Castellot, Juan Camacho, María Dolores Fernández-García, David Tarragó

**Affiliations:** 1 Centro Nacional de Microbiología, Instituto de Salud Carlos III, Majadahonda, Spain; 2 CIBER Epidemiology and Public Health (CIBERESP), Madrid, Spain; Children’s National Hospital, George Washington University, UNITED STATES

## Abstract

**Introduction:**

Meningoencephalitis in children poses a diagnostic challenge, as etiology remains unknown for most of patients. Viral metagenomics by shotgun sequencing represents a powerful tool for investigating unknown viral infections related to these cases.

**Patients and methods:**

In a two-year, reference-centre, retrospective study, we investigated the usefulness of viral metagenomics of cerebrospinal fluid (CSF) for the diagnosis of viral infectious meningoencephalitis in forty seven pediatric patients, forty of them previously tested negative with a routine neurologic panel of viral targets that included herpesvirus 1–3 and enterovirus. We enhanced the detection by targeting viral sequences by hybrid capture. Raw sequence data was analysed using three bioinformatics pipelines.

**Results:**

Out of forty remaining children with meningoencephalitis of unknown viral etiology, a significant detection of viral nucleic acid by shotgun sequencing was found in twenty one, which was confirmed in ten of them by specific PCR: seven human endogenous retrovirus K113 (HER K113), one parechovirus 3, one human herpesvirus 5 (HHV5); one enterovirus B (Echovirus 9). The remaining eleven CSF were not confirmed by PCR: three rotavirus, one human herpesvirus 7 (HHV7), one influenza A, one mastadenovirus C, one sindbis virus, one torque teno virus, one human immunodeficiency virus 1 (HIV-1), one human alphaherpesvirus 3 (HHV3), one human alphaherpesvirus 2 (HHV2).

**Conclusions:**

Underutilization of currently available meningitis-encephalitis diagnostic techniques such as BioFire® FilmArray® is the main cause of undiagnosed cases of meningoencephalitis. However, in this study we detected uncommon viruses that should be considered, including virus, rotavirus, sindbis virus, influenza A virus and HHV7. No other viral sequences that could be readily linked to CNS inflammation were detected. Some findings may stem from reagent or sample contamination, as seen with papillomavirus; for others, the clinical relevance of the virus remains uncertain and should be substantiated by further studies, as is the case with endogenous retrovirus K113 virus. Online bioinformatics pipeline CZID represents a valuable tool for analysing shotgun sequencing data in cases of neurological conditions with unknown etiology. Altogether, this study highlights the potential of shotgun sequencing in identifying previously unknown viral neuropathogens and sheds light on the interpretation issues related to its application in clinical microbiology.

## Introduction

Meningoencephalitis is a leading global cause of central nervous system (CNS) disability and mortality in children [1]. Current diagnostic workflows rely on the physician formulating a differential diagnosis based on a patient’s history, clinical presentation, and imaging findings. These are followed by serial targeted testing methods such as PCR assays for specific viruses. However, all these methods are clearly inefficient, as the cause for acute meningoencephalitis remains unidentified in up to 40–60% of patients [2, 3]. This traditional approach is particularly challenging, because these methods are constrained by their ability to detect only known viruses or a predefined panel of viral targets. Therefore, they may fail to identify novel or unexpected viral pathogens. In addition, the limited availability and volume of cerebrospinal fluid (CSF) in children owing to the requirement for lumbar puncture often compromises the number of PCR tests being performed.

Viral metagenomics by shotgun sequencing, on the other hand, involves unbiased sequencing of all nucleic acid in a clinical sample, followed by computational classification of reads to identify potential pathogens [4, 5]. This approach allows for the detection of both expected and unexpected pathogens, requiring further clinical interpretation by virologists, clinicians and bioinformaticians.

In 2015, the FDA approved the BioFire FilmArray meningitis-encephalitis (FA-ME) multiple RPC panel for use, which includes fourteen detections, seven of them viral pathogens: cytomegalovirus (CMV), enterovirus (EV), herpes simplex virus type 1 (HSV-1), herpes simplex virus 2 (HSV-2), human herpes virus type 6 (HHV-6), human parechovirus and varicella virus zoster (VZV). It has a sensitivity and specificity greater than 90% [6] and detects 90% of the viral causes of meningoencephalitis [7]. Despite having such a tool, evaluation of this clinical syndrome in the clinical setting remains variable and continues to pose a diagnostic and management challenge for clinicians.

The purpose of this study was to detect viruses not included in the currently routine PCR assays used in the diagnosis of meningoencephalitis and to describe a metagenomics method that can be applied in various clinical settings when a viral pathogen is suspected but not detected by traditional methods.

## Materials and methods

### Patients and clinical samples

In this retrospective study, 47 CSF samples from 47 children with aseptic meningitis and/or encephalitis (age ≥1 day new-born to 6 years and 2 months old), 40 of them with unknown etiology after routine clinical tests, were submitted to National Center for Microbiology (CNM) from hospitals across the country between January 2018 and July 2022, to undergo viral metagenomics by shotgun sequencing. Negative controls included six water samples. Inclusion criteria for aseptic meningitis: fever, headache, neck stiffness or bulging fontanelle, with/without altered mental status and CSF pleocytosis. Inclusion criteria for encephalitis: Main criterion (mandatory): Patients with altered mental status (defined as decreased or altered level of consciousness, lethargy) with a duration of ≥24 h. Minor criteria (2 required): fever ≥38°C within 72 h before or after presentation; Seizures; neurological focality; CSF leukocyte count ≥5/cubic mm; alteration in neuroimaging suggestive of encephalitis; alteration in the electroencephalogram compatible with encephalitis. Exclusion criteria: Absence of informed consent or not having surplus for the volume for NGS analysis (200 ul). The age of the patients and their diagnoses can be found in S1 Table. This study was approved by the Ethics Committee of the “Instituto de Salud Carlos III” CEI PI46_2017-v2_Enmienda_2020. Data were accessed for research purposes in January 2023. Authors had not access to information that could identify individual participants during or after data collection.

### Shotgun sequencing

Sequencing was performed using a pan viral (DNA and RNA viruses) metagenomic approach as previously reported [8].

#### Sample processing

Total nucleic acid was extracted from 47 clinical samples using the QiAmp mini elute virus spin kit (Qiagen, Hilden, Germany) with no RNA carrier. Six negative controls (lab grade water) representing each round of extraction procedure were used. These controls were further subjected to the complete shotgun sequencing protocol described here.

#### Library preparation

Library preparation was conducted using NEBNext® Ultra II Directional RNA Library Prep Kit for Illumina® (New England Biolabs, Ipswich, Massachusetts, USA), according to manufacturer’s protocol. Previous studies carried out in our laboratory [8] indicated that active infection caused by DNA virus were detectable through an mRNA shotgun sequencing protocol. This approach allows for the identification of viral gene expression and the precise determination of the viral pathogen responsible for the active infection. Nucleic acid was quantified by QuantiFluor® dsDNA System (Promega Madison (WIS) USA) and quality verified by Bioanalyzer High Sensitivity DNA Analysis System (Agilent Technologies, Inc. Santa Clara (CA), USA). Samples underwent further processing only when they met the manufacturer’s recommended criteria in terms of size, quality and quantity.

#### Viral nucleic acid enrichment by hybrid capture

Twist Target Enrichment Standard Hybridization v2 (Twist Bioscience, South San Francisco (CA), USA) was used following manufacturer’s protocol. The Twist Target Enrichment protocol was employed to generate viral-enriched DNA libraries for sequencing on Illumina next-generation sequencing (NGS) systems. This method is based on hybridization probes and covers reference sequences for 3,153 viruses, including 15,488 different strains.

#### Sequencing

DNA of the captured libraries was quantifed and quality verified for sequencing by QuantiFluor® dsDNA System (Promega Madison (Wis), USA) and Bioanalyzer High Sensitivity DNA Analysis System (Agilent Technologies, Inc. Santa Clara (CA), USA), respectively. Shotgun sequencing of the captured libraries was performed using a Nextseq 500 Illumina platform (Illumina Inc. San Diego (CA), USA) and ran with a NextSeq 500/550 High Output Kit v2.5 (300 Cycles), paired-end: R1:150; R2:150.

#### Data analysis and identification of virus

Sequencing raw data were processed and analysed using three different bioinformatics pipelines: CHAN ZUCKERBERG ID (CZID, formerly IDSEQ, https://czid.org/ applying a background model using 6 negative controls; Viral Discovery by PIKAVIRUS (developed at the Bioinformatic Unit, National Center for Microbiology) https://github.com/BU-ISCIII/PikaVirus; and Genome Detective. In all of these pipelines, the analysis involved multiple steps, including quality control, removal of human and non-relevant sequences, assembly of viral genomes or contigs, and the identification of viral sequences through sequence alignment and comparison to known viral databases.

#### Viral PCR confirmation

Viral pathogens detected by shotgun sequencing were confirmed by specific PCR tests, where available: Echovirus 9 by RT-PCR typing as described by Cabrerizo M et al. [9]. Parechovirus 3 by HPeV RT-PCR typing as described by Harvala H et al. [10]. Multiplex real-time PCR for cytomegalovirus (HHV5) and human herpesvirus 7 as previously described by Recio V et al [11]. The presence of K113 was confirmed using a PCR assay described by Moyes DL et al. [12]. PCR of Rotavirus by PCR as described by Mijatovic-Rustempasic S et al. [13]. Sindbis virus by a real-time RT- PCR as described by Sane J et al. [14]. Influenza A virus by PCR as described by Ruis-Carrascoso G et al. [15]. HHV2 and HHV3 by a Multiplex RT real-time PCR routinely used in CNM laboratory. Detailed information on this PCR can be found in S2 Table.

## Results

Fifty-three samples were analysed by shotgun sequencing, consisting of forty-seven CSF samples from children with meningoencephalitis and six negative controls. Among the forty-seven patients, seven cases, including five with enterovirus and two with human herpesvirus 6 were used as positive controls. Among the forty remaining children with meningoencephalitis of unknown viral etiology, significant detection of viral nucleic acid was found in twenty-one cases through shotgun sequencing. Out of these, ten were confirmed by specific PCR: seven HER K113, one parechovirus 3, one HHV5; one enterovirus B (Echovirus 9). The remaining eleven CSF samples were not confirmed by PCR: three rotavirus, one HHV7, one influenza A, one mastadenovirus C, one sindbis virus, one torque teno virus, one HIV-1, one HHV3, one HHV2. The viruses found by each bioinformatics pipeline are detailed in Table 1.

**Table 1:** Comparative data obtained by Genome Detective, Chan Zuckerberg ID and PiKAVIRUS pipelines.

ID	^#^Multiplex PCR	GENOME DETECTIVE	Chan Zuckerberg ID	PIKAVIRUS	^PCR pos-SGS
**1**	negative	negative	negative	negative	-
2*	Human herpesvirus 6	Human herpesvirus 6	Human herpesvirus 6	negative	+ control HHV6
3*	Human herpesvirus 6	Human herpesvirus 6	Human herpesvirus 6	Human herpesvirus 6	+ control HHV6
**4**	negative	negative	negative	negative	-
**5**	negative	Rotavirus A	Rotavirus A	negative	negative
**7**	negative	negative	negative	negative	-
**10**	negative	negative	Human alphaherpesvirus 2	Gammapapillomavirus 15	negative
**12**	negative	negative	negative	negative	-
**13**	negative	HER K113	negative	HER K113	HER K113
**14**	negative	HER K113	negative	HER K113	HER K113
**15**	negative	negative	negative	negative	-
**18**	negative	negative	negative	negative	-
**19**	negative	negative	negative	negative	-
**20**	negative	negative	negative	negative	-
21*	Enterovirus	Enterovirus B	Enterovirus B	negative	+ control Enterovirus
**23**	negative	negative	negative	negative	-
**24**	negative	Human betaherpesvirus 7	Human betaherpesvirus**7**	negative	negative
25*	Enterovirus	Enterovirus B	Enterovirus B	HER K113	+ control Enterovirus and HER K113
**27**	negative	Parechovirus A	Parechovirus A	Parechovirus A	Parechovirus 3
**28**	negative	negative	negative	negative	-
**30**	negative	negative	negative	negative	-
**31**	negative	negative	negative	HER K113	HER K113
**32**	negative	HER K113	negative	HER K113	HER K113
**35**	negative	HER K113	negative	HER K113	HER K113
**36**	negative	negative	Human alphaherpesvirus3	negative	negative
**38**	negative	negative	negative	negative	-
**39**	negative	Rotavirus A	negative	Rotavirus A	negative
**40**	negative	negative	negative	negative	-
**41**	negative	negative	negative	negative	-
**43**	negative	negative	Human mastadenovirus C	negative	negative
**44**	negative	Influenza A virus	Influenza A virus	negative	negative
**45**	negative	Human betaherpesvirus 5	Human betaherpesvirus 5	negative	Human betaherpesvirus 5
**46**	negative	Enterovirus B	Enterovirus B	negative	Echovirus 9
**47**	negative	HER K113	negative	HER K113	HER K113
**48**	negative	negative	negative	negative	-
**49**	negative	negative	negative	negative	-
**50**	negative	negative	negative	HER K113	HER K113
**51**	negative	negative	negative	negative	-
**52**	negative	negative	negative	negative	-
53*	Enterovirus	Enterovirus B and HER K113	Enterovirus B	Enterovirus B and HER K113	+ control enterovirus B77
**54**	negative	negative	Sindbis virus	negative	negative
56*	Enterovirus	HER K113	Enterovirus B	HER K113	+ control enterovirus and HER K113
**57**	negative	Torque teno virus	Torque teno virus	Torque teno virus	negative
58*	Enterovirus	Enterovirus B	Enterovirus B	Enterovirus B	+ control Enterovirus B77
**59**	negative	Rotavirus A	Rotavirus A	negative	negative
**60**	negative	Human immunodeficiency virus 1 and Rotavirus A	Human immunodeficiency virus 1 and Rotavirus A	negative	negative
**61**	negative	negative	negative	negative	-

ID* were positive controls; ^#^Multiplex PCR: Previous PCR screening; ^PCR pos-SGS: PCR to confirm shotgun sequencing results.

All positive controls were unambiguous determined by Genome Detective and CZID, while Pikavirus only detected viral nucleic acid from enterovirus or human herpesvirus 6 in three of them. Regarding the forty samples from children with meningoencephalitis of unknown etiology, in 67.5% (27/40), 62.5% (25/40) and 70.5% (29/40) of cases, the virus was not identified by CZID, Genome Detective, and Pikavirus, respectively. Of note is that CZID did not detected HER K113 in any sample presumably because this virus is considered a part of human genome.

CZID pipeline allowed the establishment of a background model using the six negative control samples included in the project, significantly facilitating the interpretation of the results. Additionally, the data are shown as rPM (reads per million) which helps to threshold the significance of the viral reads and provides comprehensive results to the scientist community. Taking into account the background model and the lowest rPM reached by a positive control, a threshold of 1710 rPM was stablished in this project. A Z-score different from 100 means that the virus was also found in negative controls, as in the cases of human mastadenovirus C and human betaherpesvirus 5 for samples 43 and 45, respectively. Other viruses found in negative controls with very low rPM included various papillomavirus and parvovirus NIH-CQV. Detailed parameters obtained by Chan Zuckerberg ID are shown in Table 2.

**Table 2:** Shotgun sequencing data analysed by Chan Zuckerberg ID pipeline.

ID	TAXON	SCORE	Z SCORE	rPM	R	Contig	Contig r	%id	L	E value
2*	Human betaherpesvirus 6A	63,052,756	100	1,717.8	3,063	0	0	99.2	148.8	10^−87^
3*	Human betaherpesvirus 6A	948,309,323	100	47,292.9	491,020	32	282,726	99.7	242.4	10^−154^
**5**	Rotavirus A	655,442,960	100	28,809.9	37,704	0	0	98.8	120.0	10^−67^
**10**	Human alphaherpesvirus 2	24,858,434	100	1,245.6	1,828	0	0	99.3	80.7	10^−42^
21*	Enterovirus B	1,544,915,221	100	95,595.7	171,450	8	158,152	93.4	306.6	10^−137^
**24**	Human betaherpesvirus 7	13,400,170	100	670.3	3,867	0	0	99.4	119.8	10^−68^
25*	Enterovirus B	2,303,065,968	100	115,153.3	398,406	4	397,819	88.2	2,265.7	10^−307^
**27**	Parechovirus A	8,446,795,184	100	422,339.8	121,372	2	121,371	98.6	5,317.5	10^−308^
**36**	Human alphaherpesvirus 3	174,116,687	100	8,705.8	8,788	0	0	99.4	146.2	10^−86^
**43**	Human mastadenovirus C	1,563,981,420	71.6	152,809.8	52,422	0	0	99.5	148.4	10^−88^
**44**	Influenza A virus	1,077,259,731	100	58,409.2	65,209	1	36,998	99.1	99.5	10^−47^
**45**	Human betaherpesvirus 5	35,152,428	29.5	21,763.7	25,446	1	3,296	99.5	158.1	10^−90^
**46**	Enterovirus B	970,886,532	100	58,581.7	41,946	3	8,864	92.5	177.9	10^−78^
53*	Enterovirus B	2,025,781,270	100	101,289.1	320,856	2	320,854	88.90	4,535.1	10^−308^
**54**	Sindbis virus	37,464,782	100	1,874.0	1,180	0	0	98.1	93.9	10^−49^
56*	Enterovirus B	194,928,941	100	15,876.3	61,716	0	0	96.5	147.7	10^−81^
**57**	Torque teno virus	6,496,447	100	625.6	22,794	2	15,645	87.5	367.6	10^−101^
58*	Enterovirus B	379,999,186	100	27,784.0	457,614	6	449,721	92.8	1,675.7	10^−296^
**59**	Rotavirus A	76,167,057	100	3,808.6	6,942	0	0	98.8	151	10^−88^
**60**	Human immunodeficiency virus 1	3,090,542,508	100	134,177.7	279,575	0	0	99.2	150.3	10^−88^
**60**	Rotavirus A	198,203,317	100	9,910.6	20,650	0	0	98	151	10^−86^

ID* were positive controls; **ID**: Identification of the clinical sample. **Taxon**: Best matching virus found. **Score**: Experimental ranking score used to prioritize microbes based on their abundance within the sample (rPM) and their comparison to control samples (Z-score). **Z Score**: Statistic used to evaluate the prevalence of microbes in the sample as compared to background contaminants. **rPM**: Number of reads aligning to the taxon in the NCBI NR/NT database, per million reads sequenced. **Contig**: Number of assembled contigs aligning to the taxon. **Contig** r:Total number of reads across all assembled contigs. **%id**: Average percent-identity of alignments to NCBI NT/NR. **L**: Average length of the local alignment for all contigs and reads assigned to this taxon. **E value**: Average expect value (e-value) of alignments to NCBI NT/NR. Chan Zuckerberg ID - Detect & Track Infectious Diseases (czid.org)

## Discussion

The shotgun sequencing RNA method enhanced by hybrid capture proved to be a successful approach in detecting both RNA and DNA viruses in CSF from children with meningoencephalitis of unknown etiology. Therefore, compared to multiplex real-time PCR for HHV1-2, VZV and enterovirus routinely used in CNM for diagnosis of viral meningoencephalitis, the methodology described in this study yielded twenty additional putative virological diagnosis to the seven previously obtained through PCR. In comparison to the clinically available assay “BioFire FilmArray meningitis-encephalitis (FA-ME)”, which is able to detect seven common viral pathogens causing CNS infections, sixteen additional diagnosis were obtained, including three cases of rotavirus, one of HHV7, one of influenza A, one of mastadenovirus C, one of sindbis virus, one of torque teno virus, one of human immunodeficiency virus 1, and seven of HER K113. A recent study, conducting diagnostic test accuracy meta-analysis (including sensitivity and subgroup analyses) concluded that sensitivities for HSV-1 were suboptimal and that the FA/ME test seems to be an excellent tool for ruling in but very limited for ruling out CNS infections [16]. Our data reflect a substantial improvement in the virological diagnosis of meningoencephalitis and highlight both the benefits and limitations of this approach. It allows for the identification of unexpected viruses in clinical samples, which can be crucial for understanding the patient’s condition.

In this study, we have tackled several challenges related to the use of shotgun sequencing to identify pathogens in pediatric cases of meningoencephalitis of unknown origin. First, we have streamlined the methodology by applying a unified protocol for both DNA and RNA viruses. It is important to note that this approach is particularly effective for detecting viruses that are actively replicating and in the active phase of infection, as it targets the RNA molecules they produce and positive-strand RNA viruses. Second, the hybrid capture enrichment method used covered reference sequences for 3,153 viruses, including 15,488 different strains. Thus, the system can generate enriched libraries spanning all known viral lineages. Third, we employed three distinct bioinformatics pipelines to analyse our metagenomics data and we compared their results to ensure the accuracy and reliability of our findings. Variations in results can sometimes be attributed to the specific algorithms and parameters used within each pipeline or the underlying base data. CZID pipeline introduced a background model to the project, which was proved essential for differentiating true signals from noise and contamination. It provided a reference point against which we could compare the sequences detected in our samples, allowing for more effective identification and filtration of contaminants. Introducing negative controls at the outset of each round of nucleic acid extraction was a proactive approach to contamination control. These negative controls underwent the same processing steps as our experimental samples, from nucleic acid extraction to sequencing. Negative controls contain no target DNA or RNA, serving as a baseline for detecting any contamination that may occur during sample handling, processing and driven by kit, reactive and instrumentation. Additionally, CZID pipeline expressed the normalized results of each sample in terms of rPM, Z and Z-score, which were highly valuable for result interpretation and making them readily comparable to data generated across the scientific community.

Significant samples were defined as those with a Z-score of 100 (or very close to 100) and an rPM (reads per million) above 1.717, 8. This threshold was established based on the lowest rPM value obtained from a positive control (Sample 2). Out of twenty-one cases that exhibited significant results, ten were confirmed through specific PCR testing: seven HER K113, one parechovirus 3, one HHV5 and one enterovirus B (Echovirus 9).

The presence and confirmation of endogenous K113 retrovirus by specific PCR is of potential significance because it is the only endogenous retrovirus known to produce viral infective particles [17] and it is associated with certain autoimmune diseases [12]. The clinical implications of this retrovirus in numerous pathologies are still a subject of debate, but a study by Moyes DL et al. [12] found its prevalence to be higher in multiple sclerosis and Sjögren’s syndrome. Of note is that its presence in CSF of our cohort (21.3%) was observed to be higher than in healthy children, as described in the literature. Human parechoviruses (HPeVs), currently consist of 19 different types (HPeV1-19). Parechovirus 3 is the most common viral cause of meningoencephalitis in young infants [18]. Therefore, this is a case of underutilization of FA-ME assay or Meningitis Viral 2 ELITe MGB Panel and the recently available EliTech HPeV RT-PCR Test [19]. Echovirus 9 is a well-known cause of meningoencephalitis in children [20]. This enterovirus B had not been previously detected in the CSF sample, possibly due to lower sensibility of PCR compared to shotgun sequencing or because PCR primers used in the initial screening would not bear sufficient sensitivity and capacity to amplify EV sequences of all different known genotypes [21]. However, its presence was later confirmed through specific PCR and Sanger sequencing. Reduced sensitivity due to reagent competition, and lack of flexibility to modify panel are major obstacles for the applications of the multiplex CNS pathogen detection panels in the clinical laboratory [22].

HHV5 or cytomegalovirus is one of the most clinically significant viral pathogens known to cause infections in immunocompromised patients. However, in this case, the patient was immunocompetent. Despite this, his CSF reached 21,763.7 rPM for HHV5 along with a Z-score of 29.5%, which is far from 100%. This means that HHV5 was also detected in a lower level at negative controls, possibly as a result of laboratory contamination.

In absence of confirmation through specific PCR, remaining positive identifications through metagenomics shotgun posed a challenge to explain. Therefore, they were generally regarded as contamination or artifacts: Rotavirus has been described as causing sporadic cases of meningoencephalitis, typically associated with acute gastroenteritis [23], but the three cases in our study were not associated with this condition. Therefore, the significance of this finding is uncertain, and it could be potentially attributed to contamination. Although exceptional cases of torque teno virus have been described in the literature [24], in most instances it is considered a contaminant or artifact resulting from bioinformatics processing.

Nervous system injury caused by influenza is one of the leading causes of influenza-related deaths among children, with a fatality rate of up to 30% [25]. Neurological symptoms of brain injury typically manifest on the same day or within several days after the onset of cold symptoms, with convulsions and alterations in consciousness being the most common. The common types of nervous system injury caused by influenza include influenza-associated encephalopathy (IAE) [26], Reye’s syndrome, Guillain-Barré syndrome, haemorrhagic shock encephalopathy syndrome [27], and acute necrotizing encephalopathy (ANE) [26, 28], with ANE being the most severe [28]. Our case was not associated to respiratory symptoms and its relevance was considered uncertain.

Single case reports and case series of HHV-7 related encephalitis or encephalopathy have been previously described, involving both immunocompetent and immunocompromised children and adults [29, 30]. HVS-2 and HHV3 (VZV) are common central nervous system viral pathogens, but these herpesvirus detections through shotgun sequencing could not be validated by PCR, possibly due to differences in techniques’ sensitivity.

Sindbis virus is an enveloped RNA virus widely distributed in Eurasia, Africa, Oceania and Australia. Sindbis virus is transmitted among its natural bird hosts via mosquitoes. Symptoms include fever, malaise, rash and musculoskeletal pain, and less commonly, meningitis [31]. To date, there have been no confirmed cases in Spain; therefore, this discovery may be considered uncertain.

Human immunodeficiency virus 1: It has been known to cause central nervous system alterations [32]. However, in this study, HIV was ruled out since none of the cases had a history of the virus.

Rhinovirus is a common respiratory pathogen among children throughout the year. Nevertheless, its involvement in central nervous system conditions is exceptionally rare, with only two cases reported to date: meningitis and sepsis-like illness caused by rhinovirus [33].

Regarding Mastadenovirus c, as well as other viruses like papillomavirus and parvovirus, the findings were ruled out, as their presence in negative controls is often regarded as common contaminants [34, 35].

The information obtained in this study can have significant implications for patient management, treatment strategies, and public health interventions. However, several limitations have been observed: The generation of large volumes of data can be challenging to process and analyse. The sensitivity of the method may be influenced by factors such as the amount of viral genetic material in the sample and sequencing depth. Careful sample collection, storage, and processing are essential to improve the accuracy of viral pathogen detection. Enrichment procedures, like hybrid capture (HC) used here, can be employed to increase the percentage of viral nucleic acid relative to host nucleic acid. However, enrichment methods can introduce biases, and the choice of method can impact the results. For instance, the depletion of methylated DNA to eliminate human DNA can also inadvertently remove viral DNA from viruses that rely on human machinery, such as Epstein-Barr virus (EBV). Given the potential for background reads and contamination, viral metagenomics by shotgun sequencing results should be analysed with the aid of appropriate filters. Using suitable positive and negative controls is essential for adjusting the assay threshold and evaluating the plausibility of the identified pathogens. This information emphasizes the importance of employing viral metagenomics by shotgun sequencing judiciously, taking into account its advantages and limitations, and implementing precautions to ensure accurate and meaningful results in both clinical and research settings.

Results from this study will inform ongoing efforts to transition the much-needed and promising technique of viral metagenomics by shotgun sequencing from a research tool to a routine clinical test for patients suspected of CNS infection.

## Supporting information

S1 Table Patients’ demographic and clinical information. (DOCX)

S2 Table Multiplex RT real-time PCR for HVS1-2, VZV and enterovirus. (DOCX)
