# Supplementary material for: Shotgun metagenomics to investigate unknown viral etiologies of pediatric meningoencephalitis
Source: PLoS One. 2023 Dec 21;18(12):e0296036. doi: 10.1371/journal.pone.0296036 (PMC10734945; doi:10.1371/journal.pone.0296036)
Supplement: S1 Table — (DOCX) [file pone.0296036.s001.docx]

**S1 Table: Patients’ age and Central Nervous System Disease**

| Patient Identification | Age | CNS Disease* |
| --- | --- | --- |
| 1 | 1 day | Encephalitis |
| 2 | 2 months | Aseptic meningitis |
| 3 | 1 month | Aseptic meningitis |
| 4 | 6 years, 2 months | Meningoencephalitis |
| 5 | 2 days | Aseptic meningitis |
| 7 | 8 days | Aseptic meningitis |
| 10 | 1 year, 7 months | Aseptic meningitis and ataxia |
| 12 | 1 month | Aseptic meningitis |
| 13 | 2 years, 3 months | Aseptic meningitis |
| 14 | 1 month | Aseptic meningitis |
| 15 | 1 year, 8 months | Aseptic meningitis |
| 18 | 1 year, 1 month | Aseptic meningitis |
| 19 | 3 months | Meningoencephalitis |
| 20 | 18 days | Aseptic meningitis |
| 21 | 1 month | Aseptic meningitis |
| 23 | 1 year, 11 months | Aseptic meningitis and ataxia |
| 24 | 1 month 11 days | Encephalitis |
| 25 | 7 days | Aseptic meningitis |
| 27 | 25 days | Aseptic meningitis |
| 28 | 2 months | Aseptic meningitis |
| 30 | 2 years, 2 months | Encephalitis: seizures with spasms and episodes of hypotonia |
| 31 | 4 years, 10 months | Meningoencephalitis |
| 32 | 7 days | Aseptic meningitis |
| 35 | 2 months | Aseptic meningitis |
| 36 | 7 months | Aseptic meningitis |
| 38 | 1 year, 5 months | Aseptic meningitis |
| 39 | 20 days | Aseptic meningitis |
| 40 | 7 years, 4 months | Aseptic meningitis |
| 41 | 6 days | Aseptic meningitis |
| 43 | 7 years | Aseptic meningitis |
| 44 | 2 years, 1 month | Aseptic meningitis |
| 45 | 6 months | Aseptic meningitis |
| 46 | 13 days | Aseptic meningitis |
| 47 | 10 days | Aseptic meningitis |
| 48 | 4 years, 1 month | Aseptic meningitis |
| 49 | 4 days | Aseptic meningitis |
| 50 | 3 days | Aseptic meningitis |
| 51 | 2 years, 11 months | Aseptic meningitis |
| 52 | 13 days | Meningoencephalitis |
| 53 | 6 days | Aseptic meningitis |
| 54 | 17 days | Aseptic meningitis |
| 56 | 2 years, 6 months | Aseptic meningitis |
| 57 | 3 months | Aseptic meningitis |
| 58 | 10 months | Aseptic meningitis |
| 59 | 4 years | Aseptic meningitis |
| 60 | 20 days | Aseptic meningitis |
| 61 | 25 days | Aseptic meningitis |

*Inclusion criteria for aseptic meningitis: fever, headache, neck stiffness or bulging fontanelle, with/without altered mental status and CSF pleocytosis. Inclusion criteria for encephalitis: Main criterion (mandatory): Patients with altered mental status (defined as decreased or altered level of consciousness, lethargy) with a duration of ≥24 h. Minor criteria (2 required): fever ≥38ºC within 72 h before or after presentation; Seizures; neurological focality; CSF leukocyte count ≥5/cubic mm; alteration in neuroimaging suggestive of encephalitis; alteration in the electroencephalogram compatible with encephalitis
