# Supplementary material for: Shotgun metagenomics to investigate unknown viral etiologies of pediatric meningoencephalitis
Source: PLoS One. 2023 Dec 21;18(12):e0296036. doi: 10.1371/journal.pone.0296036 (PMC10734945; doi:10.1371/journal.pone.0296036)
Supplement: S2 Table — (DOCX) [file pone.0296036.s002.docx]

S2 Table. Primers and probes used for Multiplex RT real-time PCR for detection of Human herpesvirus 1 and 2, Varicella-zoster virus and Enterovirus.

| Primer name | Sequence (5´-3´) |
| --- | --- |
| VZV | Forward: ATCGATCCATCAGCGGTCC |
|  | Reserve: CCCCGCAAGACGTTTGG |
|  | Probe VZV: *VIC*-CGATCCGAGGATTCGTA-*MGB* |
| EV | Forward: ACAIGGTGYGAAGAGYCTATTGAGC |
|  | Reverse: TGCTCCRIRGTTRGGATTAGC |
|  | Probe EV: Texas red-CCTCCGGCCCCTGAATGCG-BHQ2 |
| HSV1 | Forward: GCGGTAGGCACAAAATTCGG |
|  | Reverse: CCCCCATTGGGCTGTTG |
| HSV2 | Forward: AGCGGTATGCGCAAAATTCG |
|  | Reverse: CCCATCGGGCTGCTGG |
|  | Probe HSV 1 and 2: *FAM-*CGACAGTCGATAATC-*MGB* |
| Internal Control | Forward: CAGATTAGCAATTGGTGCGAA |
|  | Reverse: GTGGGCAAATCCGAGGAA |
|  | Probe IC: *IRD-700*-AATGATTGGGCCACGTCACG-BHQ3 |

Multiplex RT real-time PCR was performed using Quantitect Multiplex RT-PCR kit (Qiagen), following manufacturer instructions in a CFX ThermoFisher thermocycler. The cycling conditions were 50ºC for 20 minutes, 1st cycling of 6x (touchdown 0,5ºC: 94ºC for 20 seconds, 61ºC for 20 seconds, 72ºC for 20 seconds) and 2^nd^ cycling of x40: 94ºC for 30 seconds and 58ºC for 90 seconds.
